# Cystine crystal nucleation and decay in the context of cystinuria pathogenesis and treatment†

**DOI:** 10.1039/d4ra04469j

**Published:** 2024-10-10

**Authors:** Kimberley Noble, Oisín N. Kavanagh

**Affiliations:** a School of Pharmacy, Newcastle University Newcastle Upon Tyne UK Oisin.Kavanagh@newcastle.ac.uk

## Abstract

Cystinuria is a rare disease which results in the precipitation of cystine in the renal filtrate, which may cause acute kidney injury due to mechanical trauma. In this work, we attempt to explore the origin of supersaturated cystine in this context to understand disease pathogenesis. This has enabled us to reproduce the clinical habit of cystine following a comprehensive study of cystine nucleation and growth in saline, artificial and human urine. Then, we describe the physical behaviour of these crystals in the presence of: cysteamine, sodium bicarbonate, captopril, tiopronin, penicillamine, glutathione and α-lipoic acid. Surprisingly, we observe that, *in vitro*, only cysteamine and saturated sodium bicarbonate dissolve crystals at a faster rate than saline, and that when solution pH is adjusted to physiological conditions, crystal dissolution for all agents is reduced to the rate of saline alone. We highlight that the conventional hypothesis of mixed disulphide formation in cysteamine is not the fastest mechanism of cystine dissolution, but rather that cystine dissolution (in the order of hours) is dominated by pH effects. This, combined with cysteamine's ability to take part in disulfide exchange reactions may explain cysteamine's effectiveness in this condition. Overall, our findings not only contribute to an understanding of cystinuria pathogenesis but also offer insights into how we should evaluate emerging treatments.

## Abstract 2

We examine cystine crystal growth and decay in cystinuria. We identify the conditions which can reproduce classical hexagonal cystine crystals and highlight an unusual dissolution pattern.

## Graphical abstract

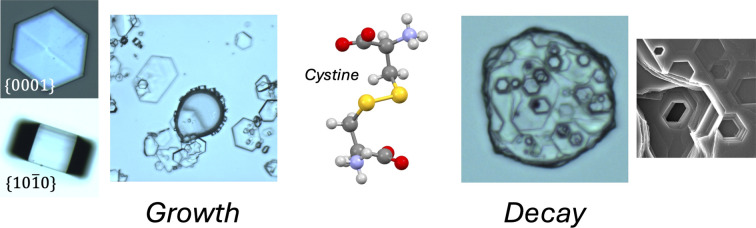

## Introduction

Precipitate induced Acute Kidney Injury (pDAKI) occurs when the solubility limit of a drug or compound is exceeded by the conditions in the kidney, and the resulting crystalline or amorphous precipitates cause renal tubular damage through mechanical trauma. pDAKI is often reported at the lowest levels in the hierarchy of evidence (as case reports) and has been described in several narrative reviews with very little mechanistic explanation.^1–3^ The focus of this study is an endogenous source of pDAKI, cystine, which is the causative species in a rare disease known more commonly as cystinuria. Cystinuria is an inherited disorder of amino acid transport that results in the failure to reabsorb cystine from the filtrate in the proximal tubule. Two genes are currently implicated, *SLC3A1* which encodes rBAT and *SLC7A9* which encodes the dibasic amino acid exchanger b0+AT. These 2 subunits form a heterodimer which allows proximal tubular reabsorption of cystine from the urine. Defects can reduce reabsorption of cystine (as well as the dibasic amino acids ornithine, lysine, and arginine) in the proximal tubule.^4^ As cystine is insoluble at physiological pH, this can lead to crystalluria and cystine containing renal stones.^5^ Although we know that cystinuria occurs due to a faulty amino acid transporter, the mechanism by which cystine supersaturation occurs remains unclear.

Cysteine is the main endogenous source of cystine as it will spontaneously oxidise. While there are some older studies evaluating thiol oxidation of cysteine in ambient conditions,^6^ little is known about oxidation rate across a range of biological conditions as much of the research explores catalysis with transition metals or acceleration with oxidants such as H_2_O_2_.^7,8^ Even less is known about the kinetics of cysteine oxidation in biological solvents such as urine. In this work we try to address this gap by presenting the kinetics of cysteine conversion to cystine in urine, exploring if supersaturation can be explained by oxidation of cysteine to cystine, large pH shifts in the filtrate, water reabsorption, or a combination of all three. We will also investigate how supersaturation conditions affect crystal nucleation and growth with the view to quantify the physiological supersaturation that enables cystine crystals with a distinct hexagonal habit to emerge.

Our second aim relates to the treatment of cystine crystalluria. In the clinic, substances which are thought to form mixed disulfides with cystine, such as cysteamine, are commonly employed. But this therapy – although effective – is very difficult for patients to take as it induces intense nausea and halitosis. This has led to the proposal of several alternative agents in ongoing and recent clinical trials which include: tiopronin (NCT03663855), SGLT2i′s (NCT04818034) and alpha-lipoic acid (NCT02910531) without a clear mechanistic basis. It is thought that many of these agents behave in the same way as cysteamine, by forming a mixed disulfide by reacting with cystine, thereby dissolving the crystals. This can be seen in a dramatic case report advocating for the use of cysteamine eye drops.^9^ Building on our first aim – which will enable us to synthesise cystine crystals with a similar habit to that seen clinically – we will assess how these agents dissolve cystine to try to investigate how they facilitate cystine dissolution relative to simpler pH mediated therapeutics such as sodium bicarbonate.

## Materials & methods

### Ellman's assay

Cysteine concentration was determined by Ellman's assay^10^ to quantify the amount of free thiol in solution. A 200 μL aliquot was diluted with 200 μL of a methanolic stock solution (1.25 mM) of 5,5′-dithiobis(2-nitrobenzoic acid) (DTNB) and 3.6 mL PBS (pH 8.0, 0.01 M). This was then analysed using an Agilent Carry 100 UV-vis spectrometer at 408 nm.

### Ethics and collection of human urine and preparation of artificial urine

Ethical approval has been granted by the Newcastle University Faculty of Medical Sciences Ethics committee (2678/41008). Fresh Human Urine (HU) was deposited at a specific time into a box and used immediately. Artificial Urine (AU) was prepared as reported in a previous publication without calcium and uric acid, which are known to precipitate.^11^

### Cysteine oxidation kinetics

Cysteine solutions of 1.65 and 3.3 mM were prepared in PBS (pH 7.4 and 6.4, 0.01 M) preheated to 25, 37.5 or 50 °C, aliquoted into 10 mL glass vials and maintained at their designated temperature by water bath until sample collection. All solutions were deoxygenated by heating and bubbling with nitrogen for 15 minutes and vials were subsequently sealed with tape to ensure that they were airtight. Samples of AU and HU were not degassed by this procedure as they were used as close to preparation/voiding as possible. As preliminary work revealed that vial headspace affected the extent of cysteine oxidation (but not the rate), it was controlled by filling vials to the brim (inverted to check for bubbles) then, a constant headspace was introduced by removing 450 μL. Free thiol content was determined using the Ellman's assay and sample vials were discarded upon opening.

### Cystine nucleation kinetics

Cystine supersaturation was induced by temperature and pH shifts. A CrystalSCAN batch reactor with turbidity and temperature probes induced temperature shifts from 51.5, 45 and 39 °C to 8 °C, at an exponential rate of −0.017 °C min^−1^, this was achieved using an oil cooling system. Larger cooling rates (−0.061 °C min^−1^) were achieved by placing the vessel into an ice bath, which reached 4.7 °C at minimum. Higher supersaturations could not be achieved by temperature shifts as preliminary work revealed that solutions of cystine are unstable beyond 60 °C. Further, decreasing the temperature close to 0 °C would nucleate ice. For induction time experiments we define *t* = 0 min as the moment at which the system becomes supersaturated (*i.e.*, when the temperature drops below the equilibrium point). We define nucleation as the moment at which turbidity was >15% over the baseline. Supersaturation in these conditions was calculated using literature temperature data.^12^

We recognise that it is more likely that pH shifts *in vivo* occur in the direction of alkaline to acid (*i.e.*, 8 to <6.8). During our preliminary work we found that it is challenging to induce supersaturation of cystine by pH shifts from alkaline to acid as high pH environments catalyse the reduction of cystine back to the high solubility cysteine.^13^ We subsequently found that 1 M HCl could dissolve up to 20 mg of cystine per mL and that this solution could be adjusted using various amounts of 5 M NaOH to the solubility minima obtained from the literature (which we consider to be 1 mM).^14^ Induction time was defined as the moment at which crystals visually nucleated, these crystals were then immediately viewed under a light microscope to assess their habit. Supersaturation is defined by eqn (1), where *C*_eq_ is the equilibrium solubility of cystine and C* is the concentration of cystine in solution at a given moment.1
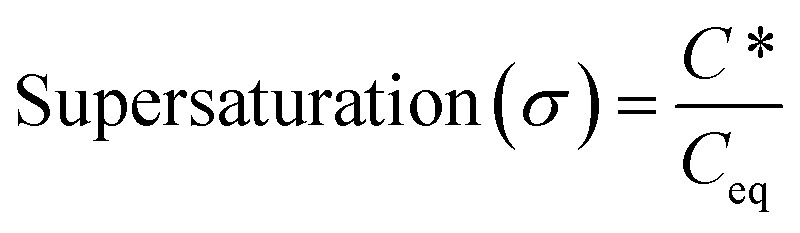


### Crystal growth and habit

Cysteine solutions (2–10 mM in PBS pH 7.4), were prepared in 10 mL glass vials in the presence of air (*via* 450 μL headspace volume) at controlled temperatures of 37.5 and 25 °C. Vials were observed daily for up to 10 days. Images of the development of the crystals were taken using a Leica DM 2700 P microscope. Sample vials were secured with a screw cap lid but were not sealed airtight. Vials were opened daily to check for crystal growth. Samples were imaged within two minutes of crystal collection to reduce effects of supersaturation by temperature drop and evaporation. Crystals generated from supersaturation experiments described above were also analysed in this way.

Micrographs of each sample were collected of various crystals after placing them onto a carbon tape and then sputter coated with gold–palladium for 90 s with a 20 mA current. Samples were then scanned at 2.5 kV with 10 mm working distance using a high-resolution field-emission electron microscope (SEM, Hitachi SU-70, Hitachi, Japan).

X-ray powder diffraction (XRPD) patterns were collected in Bragg–Brentano geometry on a PANalytical Empyrean diffractometer equipped with a sealed tube (CuKα12, *λ* = 1.5418 Å) an 1D X'Celerator detector between 5 and 40° 2*θ*. Crystals were face indexed by visualising crystal orientation by light microscopy and directly transferring this sample (in its preferred orientation) to the XRPD.

Crystal morphology was simulated using the Cambridge Crystallographic Database Centre (CCDC) Mercury software package with the Bravais Friedel Donnay Harker (BFDH) method, which predicts morphology as per inverse of the spacing between planes within the crystal (*d*_*hkl*_).^15^ Where the long *c*-axis and short *a*/*b*-axis in LCYSTI15 produces a fast growing face orthogonal to the *c*-axis. Morphology was also simulated using the VisualHabit programme where growth rate is proportional to the attachment energy of the crystal planes.^16^ Ultimately the .cif file had to be modified to produce a habit which matched empirical observations.^17^ Hexagonal cystine (C_6_H_12_N_2_O_4_S_2_) is deposited on the CSD as LCYSTI15 and belongs to the *P*6_1_22 space group with unit cell dimensions *a* = 5.42, *b* = 5.420 and *c* = 55.98 Å.

### Crystal dissolution

Cystine crystals were taken fresh from crystals grown by 6 mM cysteine in PBS (pH 7.4, 0.01 M), maintained at room temperature. Excess solution was removed from the microscope slide by paper towel, before adding drug solution. All drug solutions were prepared fresh 1 h before dissolution. Saturated drug solutions (α-lipoic acid, glutathione, penicillamine, tiopronin, captopril and sodium bicarbonate) were sonicated for at least 20 minutes in excess solid drug and stirred for at least an additional 20 minutes. Each drug was injected under the cover slip using a 29 G hypodermic needle. Additional drug solution was added periodically to prevent evaporation. Images were obtained until complete crystal dissolution or until 6 hours. ImageJ was used to determine dissolution rate by calculating crystal surface area. Further experiments were carried out with saturated drug solutions that had been pH adjusted to the values indicated in Table 2.

## Results & discussion

### Characterising cysteine oxidation kinetics

The primary source of cystine *in vivo* is from the oxidation of cysteine. We found that cysteine oxidation kinetics can be modelled by a linear function (apparent zero order, Fig. S1†) and that this rate is affected by temperature, increasing from 0.7 to 2.8% per h from 25 to 50 °C (Fig. S2†). Rate also increases from 1.47 to 3.13% per h between pH 7.4 and 6.4 in a 37.5 °C solution (Fig. S3†). The extent of the reaction can be controlled by the presence of oxygen. Although we still see some degree (*ca*. 20%) of oxidation with no headspace, increasing the headspace volume linearly increases the extent of cysteine oxidation over 10 days, but does not affect the rate (Fig. S4 and S5†). In physiologically relevant conditions, the rate of cysteine oxidation can still be modelled by a linear function where the rate increases with AU and increases further in HU (with some variation between and within individuals) (Table 1 and Fig. S6†).

**Table 1:** Kinetics of cysteine oxidation under various conditions. XTAL1a–d came from the same participant from successive voids. Mean ± SD

Conditions	Rate (% per h)
PBS (pH 6.8, 0.1 M)	1.47 ± 0.59
Artificial Urine^11^	2.71 ± 0.12

**Human Urine (HU) samples**	
XTAL1a	6.27 ± 0.17
XTAL1b	30.19 ± 0.34
XTAL1c	34.71 ± 0.47
XTAL1d	26.03 ± 0.27
XTAL6a	1.17 ± 0.23
XTAL7	4.89 ± 0.22

Taking the fastest oxidation rate (XTAL1c) and previously reported cystine solubility data in urine (*ca*. 1 mM),^14^ along with plasma and urine concentrations of cysteine, we can estimate if these rates are clinically important. If plasma cysteine is around 0.25 mM,^18^ this could produce a urine concentration between 12.5–25 mM (assuming a 50–100 fold concentrating effect). Then, if we consider the highest oxidation rate seen in our human urine samples, and a generous renal transit time of 5 minutes,^19^ it is theoretically possible to generate an additional urinary cystine of *ca*. 1 mM. When this value is placed in the context of plasma cystine (98 μM),^20^ which might be expected to concentrate to up to 9.8 mM in the urine, this suggests that cysteine oxidation in urine is unlikely to be the a major driver of supersaturation. These estimates also predict that urinary supersaturation of cystine is likely to be around 10.

### Cystine nucleation kinetics

A supersaturated solution tells us that precipitation is inevitable, but does not indicate when it might occur. It is important to understand precipitation rate in this context, as it is feasible that a supersaturated solute may exit the collecting duct (and indeed be voided) before precipitation. To evaluate this, we first generated supersaturation by rapid temperature shifts (*via* an exponentially decaying rate of −0.017 °C). From 51.5, 45 and 39 °C to 8 °C and we observe an induction time of 88 (supersaturation (*σ*) = 3.89), 117 (*σ* = 3.17) and 211 (*σ* = 2.63) minutes respectively in PBS. Using an ice bath, with an exponentially decaying rate of −0.06 °C, we find an induction time of 203 (*σ* = 4.07) mins from 49 to 4.7 °C in saline. In the ice bath with HU, across our 5 hour experiments, nucleation was not observed (*n* = 8, two participants each with four technical repeats). Although it is clear that these supersaturations are unlikely to cause cystine precipitation during renal transit, they help us to understand the supersaturations at which cystine crystals will grow, without additional nucleation. These conditions may enable cystine precipitates to grow to such a size that they block the renal tubules, which might be described as cystine stones.

**Fig. 1:**
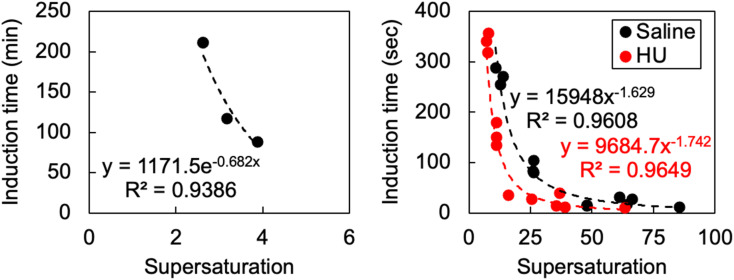
The effect of supersaturation on induction time where supersaturation was induced by temperature (left) and pH shifts (right).

As temperature cannot create a homogenous supersaturation immediately or generate large (potentially clinically important) supersaturations, we then generated supersaturation by pH shift. By adjusting the pH from *ca*. 1 to the solubility minima, we found almost instantaneous nucleation in saline and HU at supersaturations >30. We then tuned supersaturation until we extended the induction time beyond 3 minutes, which are beyond typical renal transit times. We find that induction time increases exponentially as we tune supersaturation <25, illustrated by Fig. 1, which also indicates that cystine has a critical supersaturation <30 in human urine. This behaviour is to be expected, as nucleation rate increases as a function of supersaturation. The crystal habit generated from these conditions is discussed in the following section.

pH shifts *in vivo* are likely to proceed from 4.5 to >6.8 or 8 to 4.5. As a solution completely saturated with bicarbonate produces a pH of around 8.2, and the minimum solubility of cystine according to urinary solubility data is 1 mM at pH 4.5–6.8, the theoretical maximum supersaturation by pH shift is 4 (4 mM/1 mM). In the context of our findings, considering a supersaturation of 4 did not precipitate within 5 hours in temperature shifts—confirmed by our nucleation model in Fig. 1, indicating that induction time would be expected to exceed 20 minutes—we conclude that neither pH shift or cysteine oxidation are the key mechanisms by which cystine becomes supersaturated. Clinical data has recorded urinary cystine in cystinuric patients of 3.15 mM^21^ but it typically rests between 1 and 2.5 mM.^22^ We now know that these supersaturations can be sustained for some time and that if immediate precipitation occurred, supersaturation must have approached values between 10 and 20.

### Characterising cystine crystal habit

**Fig. 2:**
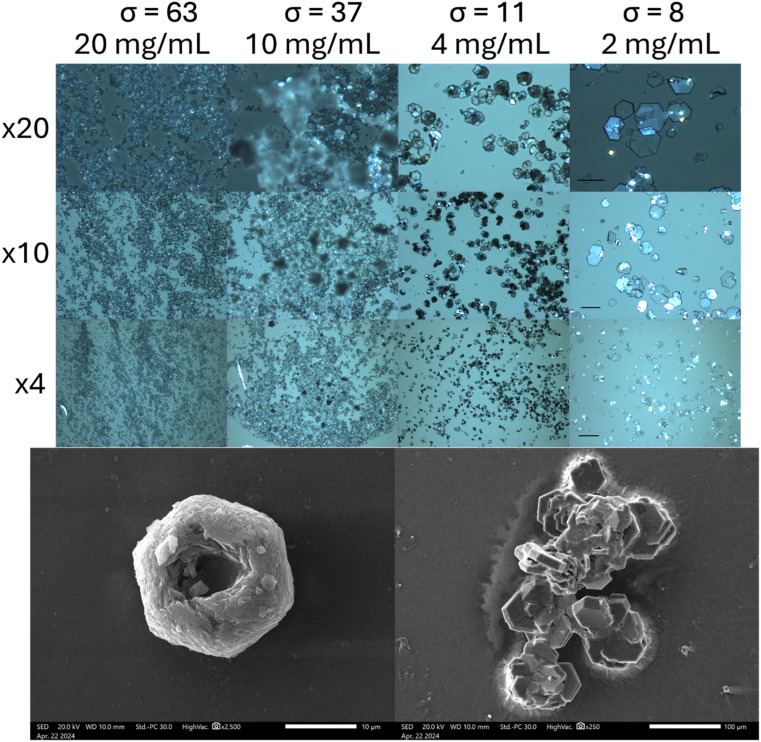
Representative microphotograph of cystine crystals which nucleate at the indicated supersaturations induced by pH shift in HU (top). A micrograph of cystine crystals which emerge from 10 mM cysteine after 3 days, which begins to mature into hexagonal plates after one day (bottom). Magnifications are inset, scales: ×20 = 50 μm; ×10 = 100 μm; ×4 = 250 μm. SEM scale = 10 μm (left) and 100 μm (right).

**Fig. 3:**
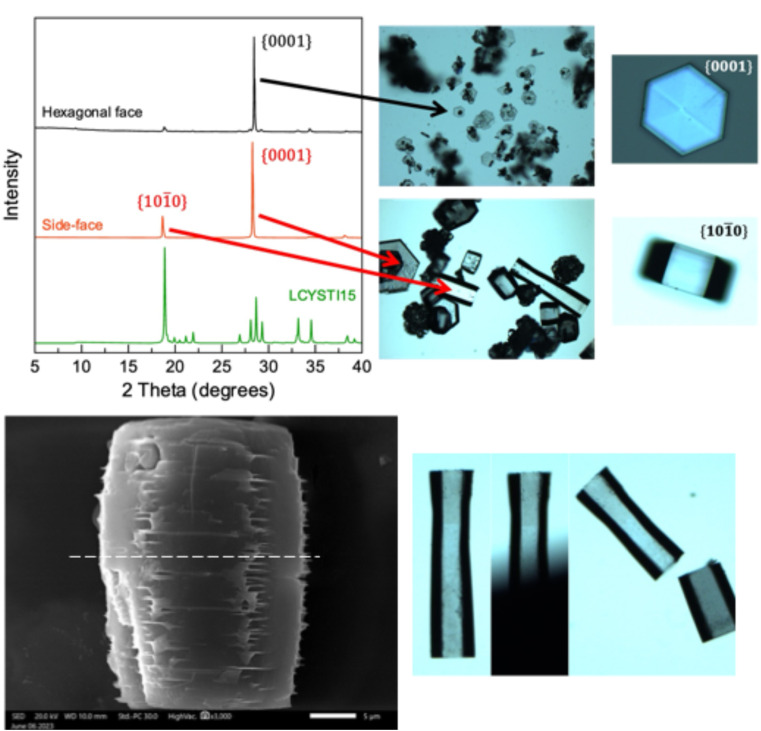
Cystine crystals face indexed by a combination of observation in a light microscope and X-ray diffraction of that preferential orientation *via* PXRD, magnification, ×4. The way in which cystine crystals grow create fractures apparent at the vertex between two {101̄0} edges, this many result in a vulnerablity to pressure normal to this face, causing cleavage. SEM scale = 5 μm.

As crystal habit is strongly influenced by the supersaturation conditions, we attempted to reproduce the “characteristic hexagonal appearance”^23^ of cystine which is seen in the clinic. When supersaturation is induced by cysteine oxidation, we find that cystine growth proceeds initially by the nucleation of hexagonally shaped platforms, on which clusters of cystine tend to nucleate by homogenous nucleation, which forms cystine rosettes emerging from a hexagonal parent crystal (Fig. 2 and S7†). Rapid supersaturation by pH shift can produce thin hexagonal cystine crystals which precipitate instantly alongside clusters of cystine crystals that have nucleated onto this hexagonal plate (Fig. 2). We find that a supersaturation of >11 produces disorganised clusters which begin to resemble clinical species after maturation, while supersaturations <11 reproducibly nucleate clinical habits immediately. Irrespective of their nucleating conditions, these cystine crystals mature over 14 days into large, thick hexagonal crystals (Fig. 2 and S7†) following Ostwald's rule. These large thick crystals and the disorganised clusters are distinct from the thin hexagonal habits which are found commonly in cystinuria samples across animal species. Neither BFDH or VisualHabit models correctly predicted cystine crystal habit, however a hybrid can be generated by modifying the .cif file (Fig. S8†). The thin hexagonal plates and thick columns exhibit different preferential orientations which were observed with light microscopy and then analysed by X-ray diffraction to assign the Miller–Bravais index's to these faces (Fig. 3). We find that the hexagonal faces can be assigned to the {0001} set and the side-faces to the {101̄0} set. Serendipitously, we found that bubbles are sometimes present in the aliquot. Cystine crystals can be observed at this liquid–gas interface and the {101̄0} faces orient perpendicularly to this interface, in the same way that these crystals rest on the glass slide (Fig. S9†). In some crystals, this homogenous nucleation appears to avoid the centre of the crystal (presumably where the dislocation spiral is) and in others, shards of cystine crystals appear to protrude from this central point. It is possible in some images to observe how successive dislocations on subsequent cystine crystals develop into these rosette shapes, with some taking the appearance of a doughnut. We have identified other reports of this hexagonal rosette habit in ZnO nanocrystals^24^ and layered double hydroxides.^25^

We also observe that these mature cystine columns with predominant {101̄0} faces appear to have many imperfections, particularly in the vertex joining two {101̄0} faces. Closer inspection of the SEM images reveal that these imperfections – characterised by thin cystine plates (≪250 nm, beyond the resolution of SEM) – protrude from this vertex as the successive hexagonal layers build up. While the thin hexagonal plates are brittle as one might expect, we thought that this irregular stacking in the mature, larger crystals, might indicate a sensitivity to shearing across the column in the [101̄0] direction. We tested this by applying pressure using a razor across the column which split the crystal evenly (Fig. 3). Tests where pressure was applied along the column and in the [0001] direction crushed the crystal irregularly. While mature cystine crystals with this habit are sensitive to shear forces in this direction, one study suggested that lithotripsy may not be sufficient to break up cystine stones which are observed clinically.^26^ This may be because cystine stones are made up of many interlocking cystine crystals as observed in the rosette habits above. However, analysis of that clinical study reveals that this conclusion was drawn after a single treatment of lithotripsy where the authors found that 37.5% of cystinuric patients were stone-free for three months as opposed to 82.5% for non-cystinuric patients, from this, they conclude a, “lack of efficacy”.^26^ We wish to emphasise that cystinuria is a genetic condition and patients are inherently different from typical (*e.g.*, uric acid/oxalate) stone formers. In other words, a single lithotripsy treatment cannot be expected to prevent further formation of cystine stones. The treatment may well have depleted existing stones, but due to the nature of the condition, could not have prevented further cystine accumulation. Indeed, the authors conclude themselves that, “cystine stones are a specific challenge that should probably be managed by combined extracorporeal and intracorporeal lithotripsy”.^26^ We provide evidence to suggest that another *in vitro* study designed to explore this hypothesis may be warranted.

Ward has previously face indexed these hexagonal cystine crystals in a series of papers using Atomic Force Microscopy, they arrived at the same Miller–Bravais index for these faces.^27^ Their elegant study provides deep insight to enable them to propose new treatment approaches which can inhibit the growth of cystine crystals with crystal growth inhibitors.^28^ They also reveal that l-cystine crystals grow in layers which are one cystine molecule thick.^27^ These layers can emerge from a dislocation in the [0001] direction, this exposes the fast growing {101̄0} faces enabling another hexagonal layer to form. Missalignment between these hexagonal layers emerging from these dislocations is sometimes visually apparent (Fig. S10†).

### Cystine crystal decay

Our second objective aims to understand how these cystine crystals behave in the presence of therapeutics which are being evaluated in clinical trials (Fig. S11†). The postulated mechanism for most of these agents (except sodium bicarbonate) is the formation of a more soluble mixed disulphide with cystine, which can enable the cystine crystals to dissolve. Sodium bicarbonate alkalises the urine and is thought to work by increasing the solubility of cystine.

**Fig. 4:**
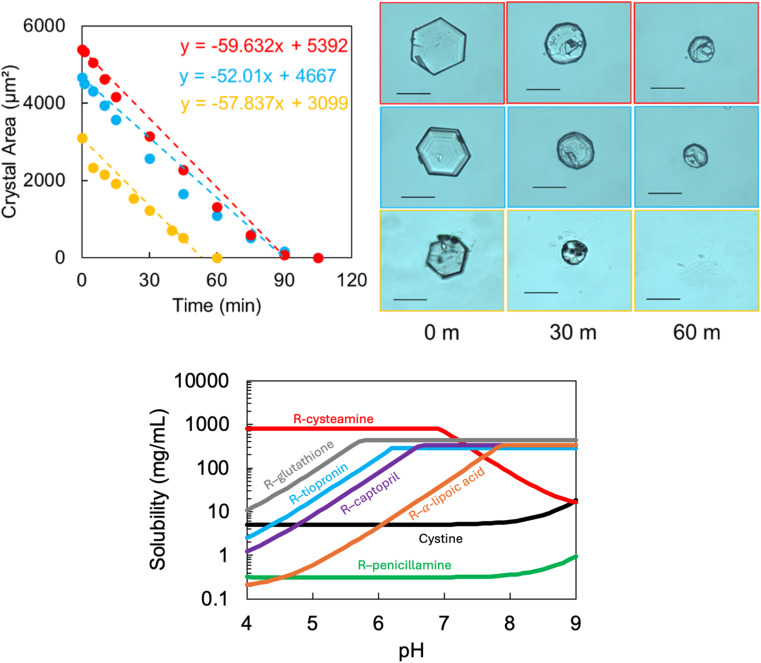
A representative example illustrating how we characterised the rate of cystine crystal dissolution in the presence of 0.38% w/v cysteamine (top). The calculated pH solubility curve for the theoretical disulfides with the thiol drugs (bottom). Magnification ×20, scale = 50 μm.

We designed an *in vitro* approach to systematically characterise the rate of cystine dissolution to try to understand (through detailed morphological assessment) how these agents interact at the crystal surface. We compare several potential and currently in use drugs: cysteamine (648 and 49.2 mM as per Cystadrops®, 0.38% w/v cysteamine) and saturated: sodium bicarbonate, captopril, tiopronin, penicillamine, glutathione and α-lipoic acid. We find that cystine dissolution can be modelled with a linear (apparent zero order) function (Fig. 4 and S12† and Table 2). Cysteamine formulations outcompete any other and only cysteamine (and sodium bicarbonate, at saturation) can dissolve the cystine crystals at a faster rate than water. Where indicated from the calculated pH-solubility curve of the mixed disulfide, the experiment was repeated in a solution close to physiologically relevant urine pH values.^11^ Surprisingly, we find that pH adjustment dramatically reduces the apparent effectiveness of cysteamine and we observe little change to the rates of the other agents.

**Table 2:** Dissolution rate of cystine crystals in the presence of saturated solutions of various free thiols. The pH of the solution is also inset

	Saturated		Adjusted pH	
	Rate (μm^2^ min^−1^)	pH	Rate (μm^2^ min^−1^)	pH
Cysteamine (5%)	192 ± 47	9.7	—	—
Cysteamine (0.38%)	56 ± 4	9.7	1 ± 1	6.9
Sodium bicarbonate	42 ± 3	8.2	—	—
Captopril	5 ± 3	2.1	3 ± 1	7.4
Tiopronin	2 ± 1	1.7	6 ± 2	7.1
Penicillamine	4 ± 4	5.1	—	—
α-Lipoic acid	4 ± 1	3.7	2 ± 2	6.7
Glutathione	2 ± 1	2.9	2 ± 2	7.1
Saline	3 ± 1	6.8	—	—

We find that rapid decay of the cystine crystal is strongly dependant on the pH of the solution for cysteamine. Paradoxically, the pH solubility plot predicts that disulfide exchange is reduced at higher pH and accelerated at lower pH values, our experimental data contradicts this hypothesis. Previous findings suggest that cystine equilibrium solubility can be increased in the presence of thiol drugs, but that cystine dissolution is heavily influenced by pH effects in clinically relevant time-frames.^29^ This might suggest that the disulfide exchange mechanism is too slow to dissolve cystine once deposited and that rapid cystine dissolution is driven by pH effects, which may be achieved by cysteamine and bicarbonate.

**Fig. 5:**
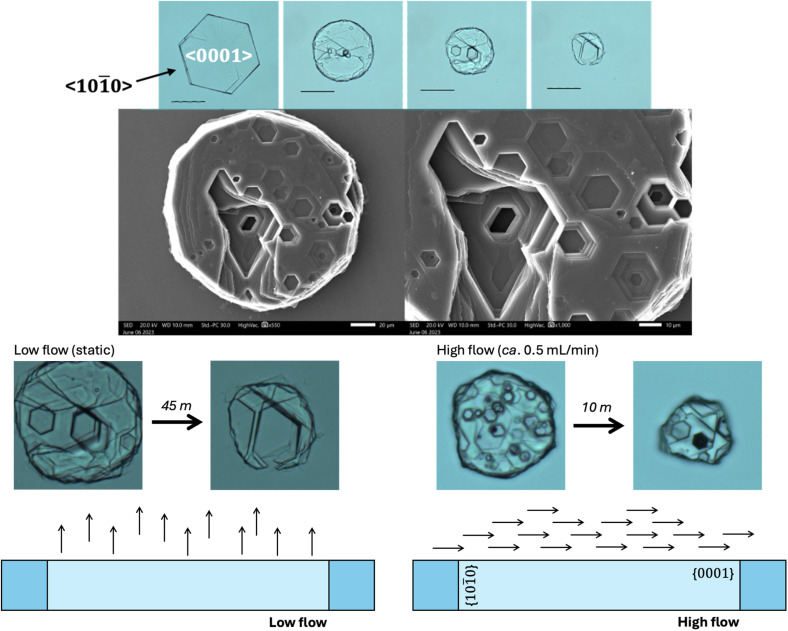
Topological appearance of cystine crystal in the presence of 5% cysteamine and a representation of the predominant flow conditions in high and low flow regimes, which alters the appearance of the cystine crystal during dissolution. Magnification ×20, scale = 50 μm. SEM magnification 20 μm (left) and 10 μm (right).

Closer inspection of the cystine crystals during our *in vitro* dissolution test reveal the presence of topologically distinct (hexagonal) pits orthogonal to the {0001} surface of the cystine crystal (Fig. 5). We also see how two denucleation points can converge to create a large pit in our static conditions. This is also observed with water but is exaggerated with cysteamine and bicarbonate. This is in contrast to previous work (in saline) that revealed that the preceding of the {101̄0} face dominates the dissolution of cystine crystals.^30^ This may be related to the experimental set-up where we have deliberately employed low flow (static) conditions as we envision that a crystallopathy may cause a reduction in flow which might change the hydrodynamics of the system.

During dissolution, the rate of decay of a solid as it dissolves is governed by surface effects and mass transport. At the surface, some reaction takes place that can be described by the following equation: rate = *k* × [reactants]^*n*^, where *k* is a proportionality constant, [reactants] is the concentration of reactants and *n* is the order of the reaction. This reaction rate might represent dissolution of the cystine solid or a series of steps involving desorption from the cystine crystal surface. As different functional groups are exposed at the various crystal surfaces, one might expect that this dissolution rate will change across the various surfaces (corresponding to a change in chemical rate). Then, once cystine dissolves into the solution, this will create interfacial layer that will become saturated with cystine prior to its movement into the bulk. This movement from the interfacial layer into the bulk can be described as mass transport and can be described mathematically by a flux equation: *J*_cystine_ = −*D*_cystine_ × ∇*c*_cystine_. Where cystine flux (*J*_cystine_) is a function of cystines diffusivity (*D*_cystine_) and the concentration gradients at a particular point in space (∇*c*_cystine_). However, the local concentration gradients are heavily dependent on convection currents in solution. These convection currents can also modify the height of this interfacial layer from which the saturated solution will diffuse into the bulk.

Ward found that <101̄0> is the dominant direction of crystal dissolution.^30^ In our study, we employ low flow conditions, where we suppose that convective flow parallel to the <0001> direction brings dissolving cystine out of the hexagonal pits and into the bulk, enabling the dissolution to create deep pits in the <0001> direction. When we increase the rate of flow we reinstate the dominance of the <101̄0> dissolution direction as we restore mass transport effects (Fig. 5).

### Implications of our findings

We conclude that while pH shifts and cysteine oxidation may contribute to urinary cystine supersaturation, renal water reabsorption is likely to be the major driver of cystine precipitation in the renal filtrate; thus, water intake is a key therapeutic consideration. We have identified that immediate clinical cystine precipitation probably emerges from supersaturations >6 and ≤11. These conditions allow us to recreate the thin hexagonal cystine crystals which are observed clinically and find that these crystals are made up of stacked hexagonal layers which are misaligned when viewed along the <0001> direction, which may make them particularly vulnerable to mechanical destruction. When we examine these crystals in the presence of therapeutic agents we find that dissolution at rates faster than saline occur only in pH environments >8.2. Therefore, we suggest that treatment of precipitated cystinuria solids should be guided by urinary pH and volume, while disulfide exchange may be more important for the prevention of cystinuria. The rates of disulfide exchange for these agents is the focus of an ongoing investigation.

In summary, we provide a nuanced understanding of cystine crystal nucleation, growth and dissolution behaviour which can guide therapeutic strategies to treat cystinuria.

## Data availability

All data will be uploaded to an Open access repository held by Newcastle University. It will also be made available to researchers on request.

## Conflicts of interest

There are no conflicts to declare.

## Supplementary Material

RA-014-D4RA04469J-s001
