# Supplementary material for: Cystine crystal nucleation and decay in the context of cystinuria pathogenesis and treatment
Source: RSC Adv. 2024 Oct 10;14(44):32063–72. doi: 10.1039/d4ra04469j (PMC11465997; doi:10.1039/d4ra04469j)
Supplement: RA-014-D4RA04469J-s001 [file RA-014-D4RA04469J-s001.pdf]

Supplementary Information (SI) for RSC Advances.
This journal is © The Royal Society of Chemistry 2024

Supplemental information

Cystine crystal nucleation and decay in the context of cystinuria pathogenesis and treatment

Kimberley Noble, Oisín N. Kavanagh*

School of Pharmacy, Newcastle University, Newcastle upon Tyne, UK.

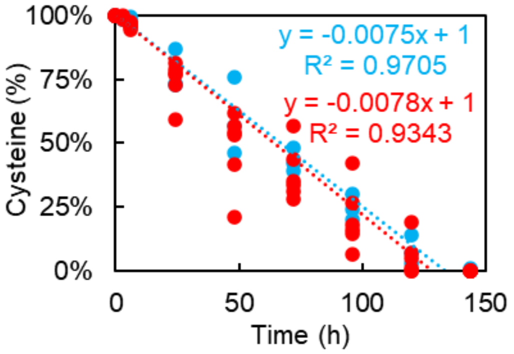

Figure S1. Conversion of 3.3 mM (red) and 1.65 mM (blue) cysteine to cystine in PBS pH 7.4 at 32°C in the presence of oxygen.

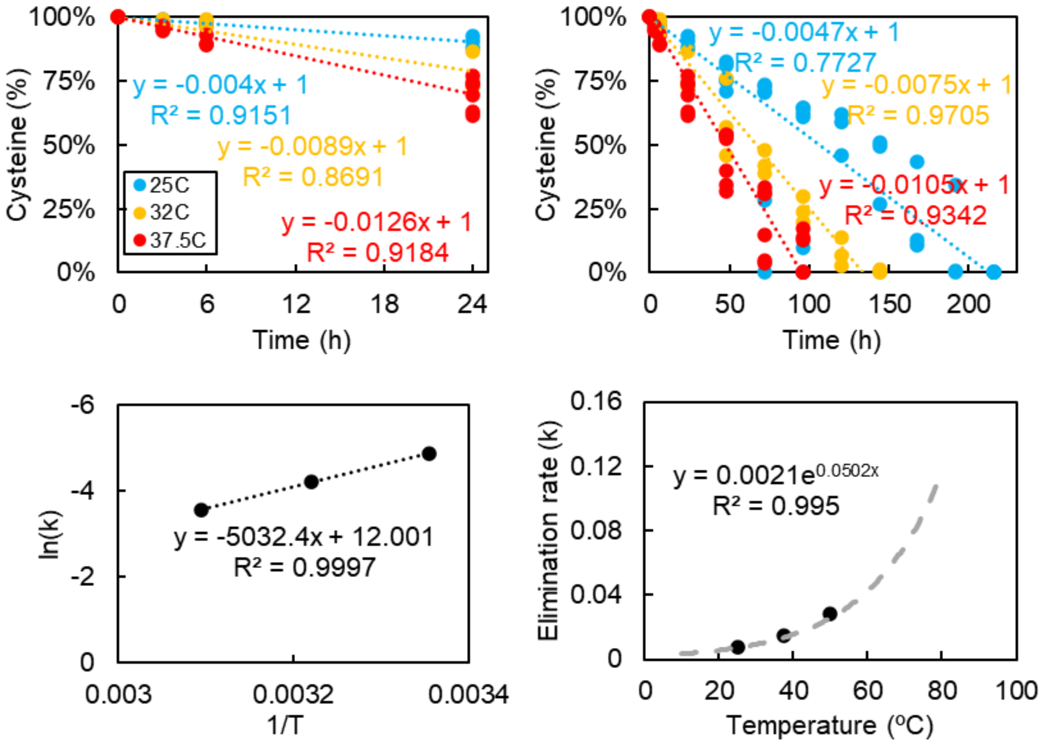

Figure S2. Conversion of 1.65 mM cysteine to cystine in PBS at pH 7.4 at controlled temperatures of 25°C (blue), 32°C (yellow) and 37.5°C (red) with the rate data converted onto an Arrhenius plot.

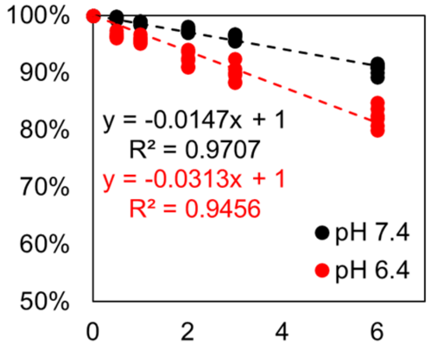

Figure S3. Elimination rate of cysteine in PBS at pH 7.4 and 6.4 at 37.5 ºC.

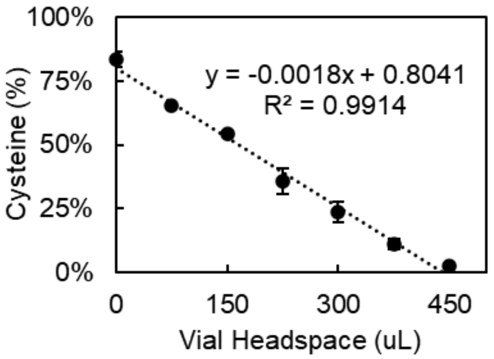

Figure S4. Extent of conversion of cysteine (1.65 mM) to cystine after 10 days, starting cysteine solution was bubbled with nitrogen to remove dissolved oxygen from solution and airtight sealed. Controlled headspace volumes of 0 – 450 µL were introduced to control oxygen availability to the system.

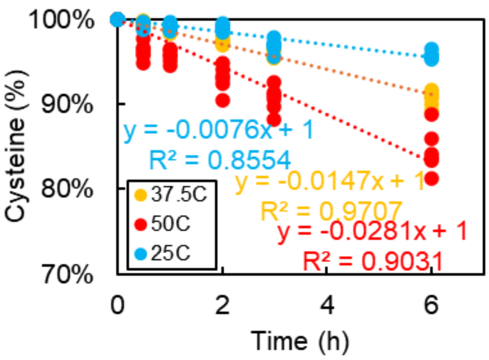

Figure S5. Conversion of 1.65 mM nitrogen bubbled and airtight sealed cysteine in PBS pH 7.4 at controlled temperatures (25°C, 37.5°C and 50°C).

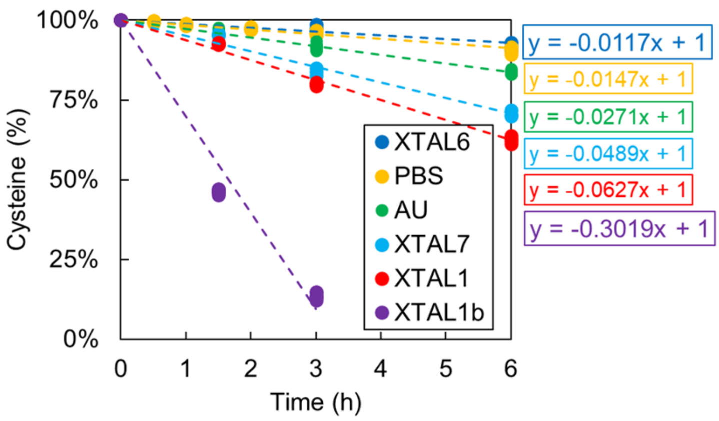

Figure S6. Elimination rate of cysteine in various conditions.

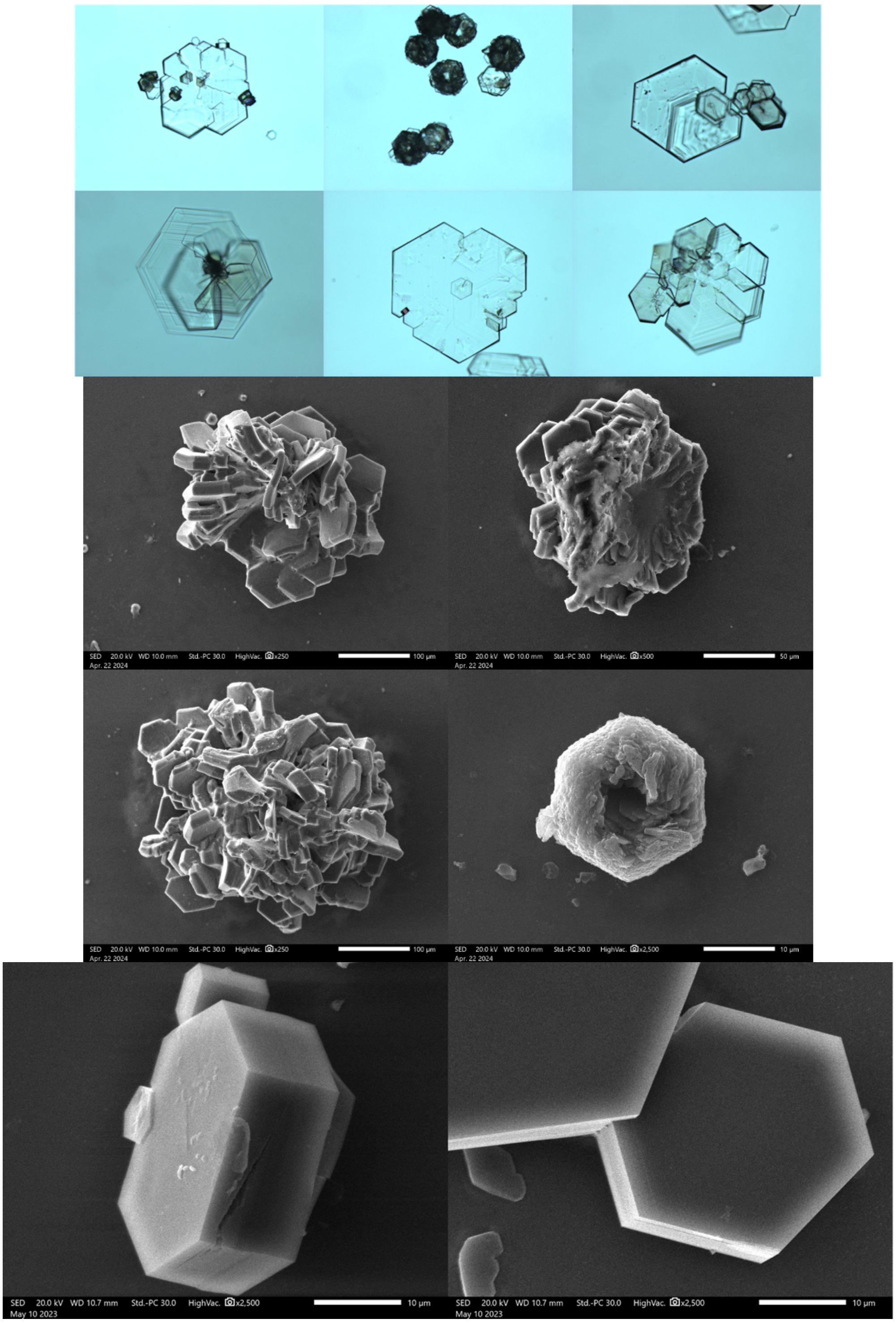

Figure S7. Micrograph of cystine crystals forming rosettes which mature over 14 days.

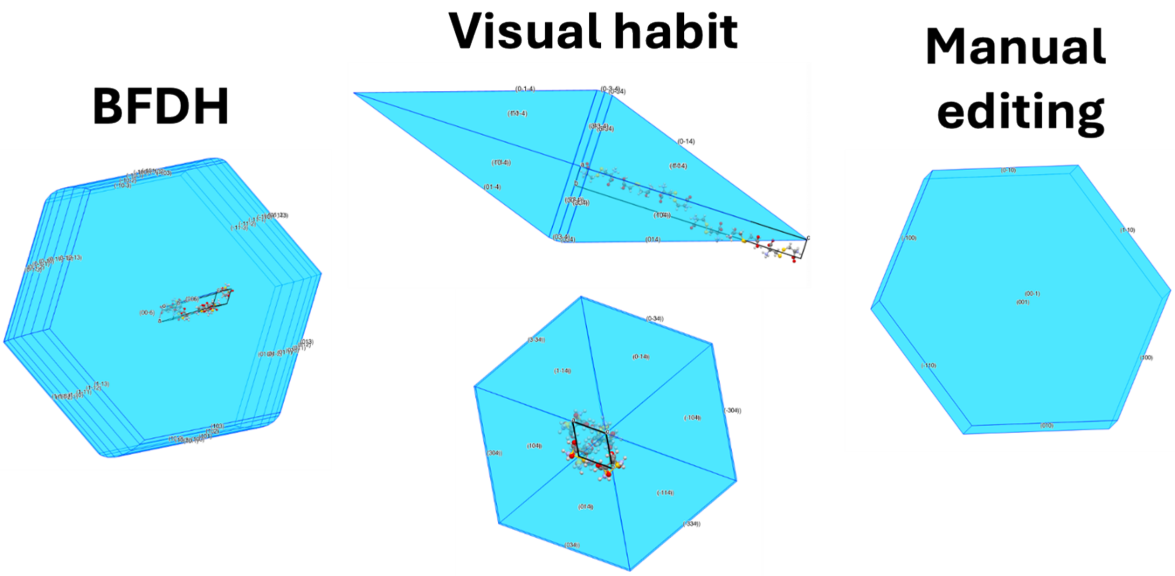

Figure S8. The predicted (and empirically modified) crystal habit of L-Cystine.

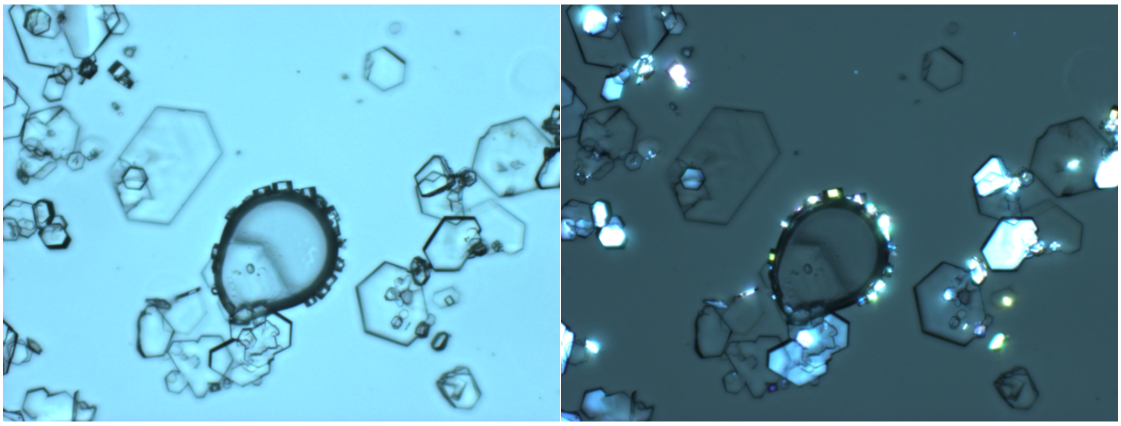

Figure S9. Anomalous cystine behaviour at the air-liquid interface.

Figure S10. Micrograph of cystine crystals forming rosettes which mature over 14 days.

Figure S11. The chemical structures of the key therapeutic agents explored in this study.

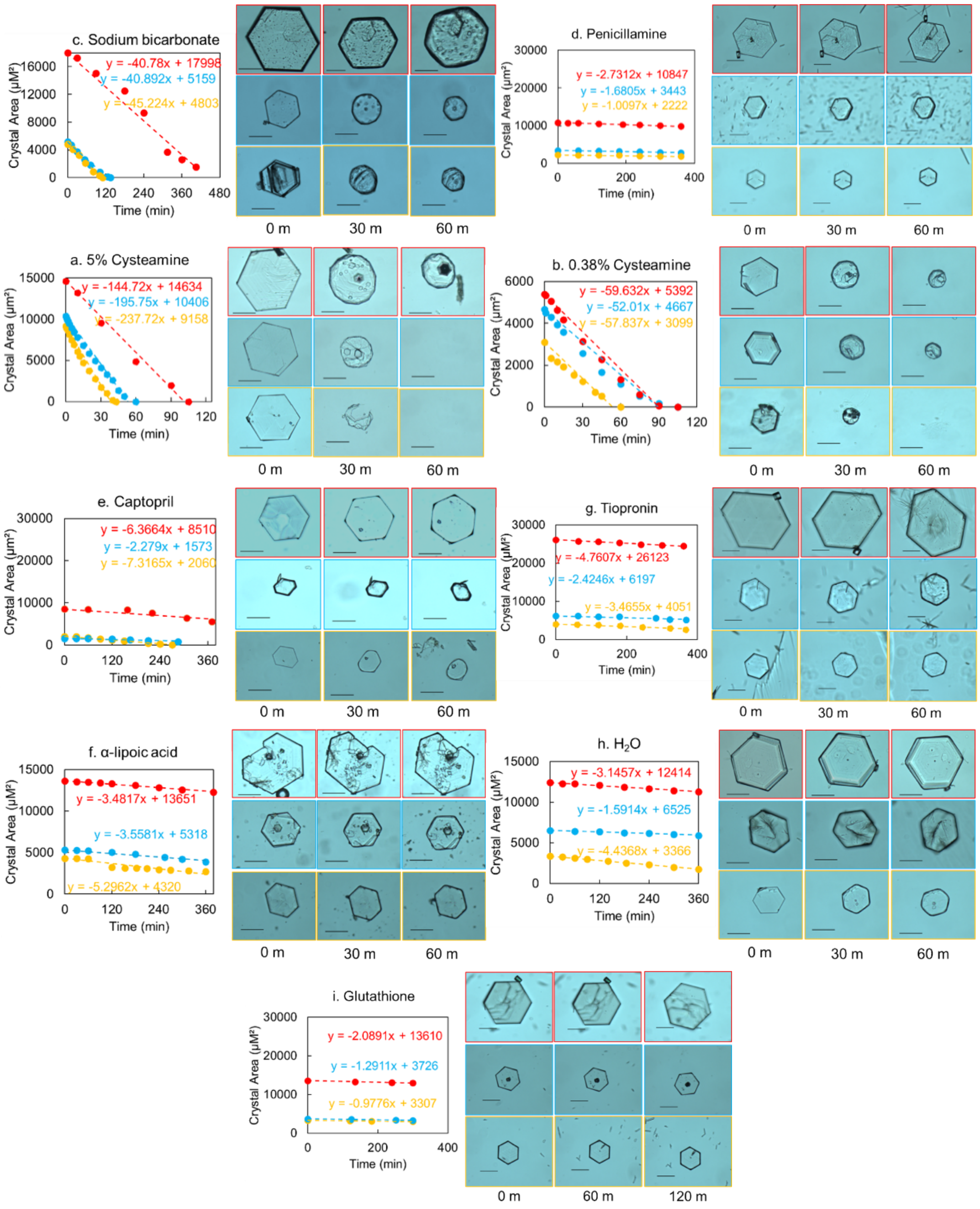

Figure S12. The rate of cystine crystal dissolution in the presence of therapeutic agents used in cystinuria. Magnification x20, scale = 50 μm.
